# Outcome Assessment According to the Thickness and Direction of the Acellular Dermal Matrix after Implant-Based Breast Reconstruction

**DOI:** 10.1155/2021/8101009

**Published:** 2021-11-16

**Authors:** Joon Hur, Hyun Ho Han

**Affiliations:** Department of Plastic Surgery, Asan Medical Center, University of Ulsan College of Medicine, Seoul, Republic of Korea

## Abstract

**Purpose:**

The acellular dermal matrix plays an important role in reinforcing thin mastectomy skin and repositioning the implant in prosthetic breast reconstruction. As the concept of prepectoral plane has become widespread, the role of the acellular dermal matrix has become increasingly important. However, evidences and standards for appropriate thickness and direction during placement remain insufficient. This study is aimed at testing the assumption that differences in the acellular dermal matrix thickness and orientation during placement may affect surgical outcomes including the incidence of postoperative complications.

**Methods:**

This was a retrospective single-centered analysis of 43 patients (50 breasts) who underwent implant-based reconstruction with MegaDerm® (L&C Bio, Seoul, Korea) and 23 patients (23 breasts) who underwent implant-based reconstruction with DermACELL® (LifeNet Health, Virginia Beach, VA, USA), two types of human-derived acellular dermal matrix. All surgeries were performed by a single surgeon. Demographic variables, surgery-related factors, and complications were compared between a thick matrix group (1.5–2.3 mm) and a thin matrix group (1.0–1.5 mm). The same processes were performed in the nonreverse and reverse matrix insertion groups.

**Results:**

Baseline demographics and surgery-related data were summarized according to matrix thickness and direction. There were no significant intergroup differences in the demographic variables such as history of smoking, radiation, or chemotherapy. The mean drain volume was significantly higher in the thick matrix group than that in the thin matrix group (*p* = 0.0445). However, there were no significant differences in overall complication rates by matrix thickness (*p* = 0.3139). Additionally, there were no significant differences in complications between the nonreverse and reverse matrix insertion groups (*p* = 0.538).

**Conclusion:**

Our findings suggest that patients with a thick acellular dermal matrix need a prolonged period for engraftment. However, the thickness did not directly affect the surgical outcomes between the thick and thin matrix groups. Likewise, the orientation in which the acellular dermal matrix was inserted did not affect the surgical outcomes including postoperative complications.

## 1. Introduction

Acellular dermal matrix (ADM) is a tissue graft processed from cadaver, animal, or synthetic materials. ADM is commonly used in prosthetic breast reconstruction for its pliability, strength, tissue integration, and potential role in the mitigation of capsular contracture [1]. Specifically, the ADM acts as a scaffold for autologous cell growth and revascularization, providing an extra layer of soft-tissue support for the prosthesis [2].

The use of ADM in breast reconstruction is gradually expanding [3, 4]. Using ADM, the thickness of the mastectomy flap can be reinforced, the position of the implant is stabilized, and complications such as capsular contracture can be reduced [5–10]. As the use of ADM increases and the concept of prepectoral breast reconstruction becomes widely accepted in prosthetic reconstruction [11, 12], more drawbacks of ADM, such as seroma and infection of the engraftment issue, have been reported [13–20]. Also, since several types of ADM are available from porcine, bovine, and human sources from different manufacturers [21], there are a variety of physical and biochemical characteristics [22], and doctors may use an ADM according to either the manufacture protocols or their own protocols based on surgical environment and experience. Therefore, it is necessary to gather data regarding the different techniques and their outcomes to standardize protocols for ADM use.

This study is aimed at describing how ADM thickness and insertion direction of two types of human-derived ADM affect implant-based breast reconstruction outcomes. We hypothesized that a thicker ADM would prolong biointegration and result in poorer outcomes in breast reconstruction. We also hypothesized that there would be differences in outcomes based on ADM insertion direction since the ADM has different anterior and posterior sides (dermal and basement, respectively).

## 2. Patients and Methods

### 2.1. Population and Study Design

A retrospective chart review was performed to identify patients who had undergone direct-to-implant breast reconstruction after mastectomy using ADM between April 2017 and March 2020 in a single center. All the surgeries were performed by a single surgeon.

**Figure 1:**
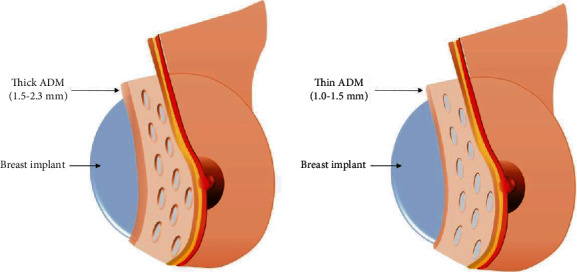
Different acellular dermal matrix thicknesses.

**Figure 2:**
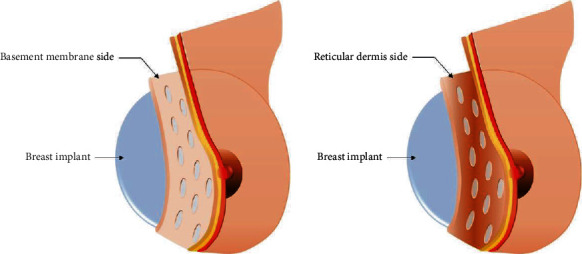
Different acellular dermal matrix insertion directions.

MegaDerm® (L&C Bio, South Korea) and DermACELL® (Stryker, USA) ADM were used in this study. The group of patients treated with MegaDerm® was divided into two subgroups by ADM thickness. The thin ADM was 1.0–1.5 mm thick, while the thick ADM was 1.5–2.3 mm thick (Figure 1). The group of patients treated with DermACELL® was divided into two subgroups by direction of insertion with the basement membrane side contacting the implant in one group and the dermal side contacting the implant in the other group (Figure 2). Preoperatively, the types of ADM used in each operation were selected randomly regardless of the thickness of the mastectomy flap or the state of flap circulation. Patients who underwent simultaneous additional or secondary procedures during the operation were excluded. Patients who underwent an implant change or expander-to-implant were also excluded from the study.

### 2.2. Data Collection

Baseline data included age, body mass index, smoking history, obesity, history of neoadjuvant radiotherapy, history of adjuvant radiotherapy, history of neoadjuvant chemotherapy, and history of adjuvant chemotherapy.

Surgery-related factors were also collected and consisted of mastectomy specimen weight, inserted implant size, ADM area, time to suction drain removal, total drainage volume, mastectomy method, implant insertion plane, implant texture, and breast cancer laterality. In the case of prepectoral implant insertions, ADM coverage was performed only at the anterior aspect of implants.

The following postoperative complications were also assessed for at least 6 months: capsular contracture, rippling, nipple sloughing, mastectomy flap necrosis, seroma, hematoma, red breast syndrome, implant rotation, and animation deformity. We defined major complications as those requiring surgical interventions and minor complications as those that did not.

### 2.3. Statistical Analysis

Mean, standard deviation, median value, and range were calculated for all continuous variables, while absolute frequencies and percentages were calculated for all categorical variables. All categorical variables were calculated and compared using the chi-squared test or Fisher's exact test, while all continuous variables were calculated and compared using the Mann-Whitney *U* test or Student's *t*-test. To compare the complication rates between groups, Fisher's exact test and generalized estimating equation model for logit link were applied. Odds ratios and 95% confidence intervals were also calculated. The statistical analyses were conducted using IBM SPSS (IBM Corp., Armonk, NY, USA). The criterion for significance was *α* < 0.05 (one-sided). The criterion for negating the preliminary differences between groups was *α* < 0.05.

## 3. Results

### 3.1. Results by ADM Thickness

Patient demographics according to ADM thickness are summarized in Table 1.

There were no significant differences between the patients in the thick and thin ADM groups regarding mean age, body mass index, smoking history, obesity, history of chemotherapy, or history of radiotherapy (Table 1). There were no significant differences between the thick and thin ADM groups in surgery-related factors including time to drain removal, operation site, mastectomy type, implant insertion plane, implant texture, or axillary lymph node dissection (Table 2). However, there was a significantly higher mean drain volume in the thick ADM group (994.73 mL) than that in the thin ADM group (723.35 mL; *p* = 0.0445).

The complications of the two groups are described in Table 3. There were no significant differences in all types of complications between the thick and thin ADM groups. Although the *p* value of the mastectomy flap necrosis rate did not indicate significance (*p* = 0.06123), the incidence of mastectomy flap necrosis tended to be higher in the thick ADM group (23%) than in the thin ADM group (4%).

### 3.2. Results by ADM Orientation

Demographics by ADM orientation are summarized in Table 4. There were no significant differences between the nonreverse and reverse ADM insertion groups in mean age, body mass index, smoking history, obesity, history of chemotherapy, or history of radiotherapy (Table 4). The mean drain volume in the nonreverse ADM insertion group was 580.24 mL, while that of the reverse ADM insertion group was 524.67 mL (*p* > 0.999). In addition, there were no significant differences between the nonreverse and reverse ADM insertion groups regarding surgery-related factors, including time to drain removal, operation site, mastectomy type, implant insertion plane, implant texture, and axillary lymph node dissection (Table 5).

In the nonreverse ADM insertion group, the minor complication rate was 7.14% (1/14) without major complications. In the reverse ADM insertion group, the minor complication rate was 22.22% (2/9), with no major complications. There were no statistically significant differences between the nonreverse and reverse ADM insertion groups in terms of complications (Table 6).

## 4. Discussion

This study is aimed at describing how thickness and insertion direction of two types of human-derived ADM affected implant-based breast reconstruction outcomes. Our results indicate that there are no significant differences in intraoperative outcomes according to thickness or insertion direction.

Since ADM was first introduced in 2001, it has become increasingly common in prosthetic breast reconstruction. Specifically, the prepectoral technique has played an important role in the accelerated ADM use in the past 3–4 years [23–25], and ADM accounts for a greater portion of surgeries compared with synthetic mesh in recent prosthetic breast reconstructions. However, a synthetic mesh remains an alternative tool to cover breast prosthetics. Some studies suggest that the use of a synthetic mesh improves aesthetic results and reduces the incidence of capsular contracture, very similar to the role of ADM but with lower cost [26]. In addition, the use of a synthetic mesh has some advantages such as reducing surgical time for implant positioning, thereby lowering exposure time and risk of infection [27]. Therefore, the current research is aimed at elucidating the effectiveness of ADM, describing the associated complications, and exploring the reasons for the increased cost [28].

Thus, whether ADM thickness affects prosthetic breast reconstruction outcomes requires consideration. Generally, a thick ADM provides robust mechanical support, but there are also some concerns with engraftment. Prolonged engraftment may increase the risk of complications like seroma or infection and may induce differentiated skin textures [13–20]. In other words, a thin ADM is more easily incorporated and less likely to cause the complications such as seroma or infection. Determining whether ADM thickness affects prosthetic breast reconstruction outcomes can provide surgeons with evidence when deciding whether to use an ADM in their surgeries.

This study revealed no significant differences in complication rates between patients with thick and thin ADM. In terms of demographic and surgery-related factors, only the mean drain volume differed between the groups. By confirming the increased Jackson-Pratt (JP) drain volume in the thick ADM group, we concluded that a thicker ADM needs a longer drain time. However, despite the increased drain volume and time to engraftment, the prosthetic breast reconstruction outcomes were not affected.

At the outset of our study, we expected that incision types in nipple sparing mastectomy would be a factor affecting outcomes. A total of three types of incisions were included in the present study, specifically, radial, periareolar, and lateral incisions. Fortunately, there was no difference in the proportion of complications by the incision type. Even though there were cases of nipple slough, no additional surgical procedures were needed. However, we could not generalize the influence of incision type due to the small numbers of cases included.

**Figure 3:**
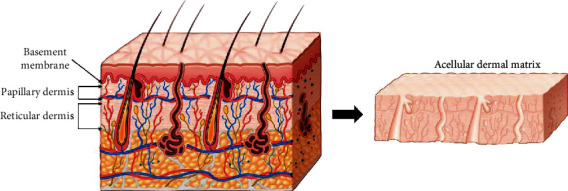
The surface side is the basement membrane, and the opposite side is the reticular dermis. After the decellularization process, only the structure of the dermis is utilized.

The two sides of ADM have different biologic characteristics [29]. The surface side serves as the basement membrane, and the opposite side contains the reticular dermis (Figure 3). Manufacturers usually instruct surgeons to place the reticular dermis side on the site to be engrafted, which means the basement membrane side faces the implant, while the reticular dermis side faces the mastectomy skin. However, it is possible to place the ADM inside out due to a surgeon's lack of experience or mistakes during surgery. Therefore, it is important to elucidate the effects of ADM insertion orientation on outcomes. If ADM insertion orientation does not affect outcomes and the role of the basement membrane is not significant, manufacturers can produce multiple sheets of ADM from a thicker dermis area such as the back or head. Therefore, determining whether the basement membrane is necessary to maintain the shape of the breast by helping to maintain the dermal strength later is important for clinical practice.

In the present study, we found no significant differences in baseline demographics, surgery-related factors, or complication rates between the nonreverse and reverse ADM insertion groups. Thus, we found no relation between ADM thickness and orientation with prosthetic breast reconstruction outcomes. It is important to note that ADM thickness and orientation did not affect engraftment. These results suggest that ADM can be used with more flexibility. For example, a thicker ADM can be applied to provide stronger mechanical support in cases of patients with very thin skin tissue, while a thinner ADM can be applied to obtain more pleasing aesthetic results in patients with a thick mastectomy flap or those undergoing a risk-reducing mastectomy.

Our study has several limitations. First, our study used a retrospective design, and a small number of patients were included. In addition, there may be a possible selection bias since all the patients were of the same race; since Asian patients tend to be slim, there was a lower possibility of including patients with large breasts. Thus, the possibility of complications may be underestimated.

## 5. Conclusion

Our results suggest that a thick ADM requires a prolonged engraftment period due to the large drainage volume. This, however, did not directly affect the surgical outcomes between patients receiving a thick versus thin ADM. Likewise, the orientation in which the ADM was inserted did not affect surgical outcomes or the incidence of complications.

## Figures and Tables

**Table 1:** Patient baseline demographics and concurrent treatments.

	Thick ADM	Thin ADM	*p* value
No. of patients	23	20	
Age (yr)			0.3538
Mean ± SD	45.65 ± 10.52	42.80 ± 9.24	
Range	25-61	24-61	
BMI (kg/m^2^)			0.112
Mean ± SD	24.48 ± 7.01	21.90 ± 2.60	
Range	17.69-50.54	17.58-27.01	
Smoking (*n*, %)	1 (4.35%)	3 (15%)	0.3235
Obesity (*n*, %)	2 (8.7%)	0 (0%)	0.4906
Radiotherapy (*n*, %)	6 (26.09%)	5 (25%)	0.9351
Preoperative	0	0	
Postoperative	6 (26.09%)	5 (25%)	0.9351
Chemotherapy (*n*, %)	10 (43.48%)	12 (60.0%)	0.2797
Preoperative	5 (21.74%)	1 (5%)	0.1918
Postoperative	10 (43.48%)	11 (55%)	0.4509
Pre+post	5 (21.71%)	0 (0%)	0.0511

ADM: acellular dermal matrix; SD: standard deviation; BMI: body mass index. Obesity was defined as BMI > 25 kg/m^2^.

**Table 2:** Surgery-related factors.

	Thick ADM	Thin ADM	*p* value
No. of breasts	26	24	
Implant size (cc)			0.176
Mean ± SD	272.462 ± 100.224	234.375 ± 95.432	
Range	160-480	95-450	
Mastectomy weight (g)			0.5999
Mean ± SD	365.208 ± 273.339	295.958 ± 161.124	
Range	125-1150	96-828	
ADM size (m^2^)			0.067
Mean ± SD	121.615 ± 33.555	109.750 ± 34.132	
Range	70-192	75-192	
Jackson-Pratt drain (mL)			0.0445
Mean ± SD	994.731 ± 539.652	723.354 ± 365.827	
Range	268-2737	320.5-1588	
Time to drain removal (day)			0.5297
Mean ± SD	19.385 ± 6.350	18.375 ± 4.735	
Range	9-31	12-27	
Operation site (*n*, %)			0.5547
Left	13 (50%)	10 (41.67%)	
Right	13 (50%)	14 (58.33%)	
Mastectomy type (*n*, %)			0.4585
NSM	17 (65.38%)	18 (75%)	
Radial incision	10 (58.82%)	11 (61.11%)	
Periareolar incision	4 (23.53%)	4 (22.22%)	
Lateral incision	3 (17.65%)	3 (16.67%)	
SSM	9 (34.62%)	6 (25%)	
ALND (*n*, %)	21 (80.77%)	19 (79.17%)	>0.999
Insertion plane (*n*, %)			0.9819
Prepectoral	12 (46.15%)	11 (45.83%)	
Subpectoral	14 (53.85)	13 (54.17%)	
Implant texture (*n*, %)			0.5791
Smooth	10 (38.46%)	13 (54.16%)	
Microtexture	15 (57.69%)	10 (41.67%)	
Macrotexture	1 (3.85%)	1 (4.17%)	

ADM: acellular dermal matrix; SD: standard deviation; ALND: axillary lymph node; SSM: skin sparing mastectomy; NSM: nipple areolar skin sparing mastectomy.

**Table 3:** Postoperative complications.

	Thick ADM	Thin ADM	OR^∗^	95% CI		*p* value
Total complication (*n*, %)			0.5352	0.1585-1.8069		0.3139
No complication	13 (50%)	16 (66.67%)				
Minor^∗^	9 (34.62%)	8 (33.33%)				
Major^∗^	4 (15.38%)	0 (0%)				
Capsular contracture	1 (3.85%)	0 (0%)	NE			
Rippling	1 (3.85%)	3 (12.5%)	3.6379	0.3450-38.3556		0.2826
Nipple sloughing	3 (11.54%)	2 (8.33%)	0.3509	0.0335-3.6744		0.3821
Rotation	1 (3.85%)	1 (4.17%)	1.2589			0.8711
Animation	0 (0%)	2 (8.33%)	NE			
RBS	1 (3.85%)	1 (4.17%)	1.0935	0.0636-18.8027		0.9509
Mastectomy flap necrosis			0.1288	0.0146-1.1371		0.0651
No complication	20 (76.92%)	23 (95.83%)				
Minor	2 (7.69%)	1 (4.17%)				
Major	4 (15.38%)	0 (0%)				
Seroma			0.5259	0.0438-6.3136		0.6123
No complication	24 (92.31%)	23 (95.83%)				
Minor	2 (7.69%)	1 (4.17%)				
Major	0 (0%)	0 (0%)				
Hematoma			NE			
No complication	26 (100%)	24 (100%)				
Minor	0 (0%)	0 (0%)				
Major	0 (0%)	0 (0%)				

^∗^Major: the complications which needed secondary surgical procedures. ^∗^Minor: the complications which did not need secondary surgical procedures. ADM: acellular dermal matrix; OR: odds ratio; CI: confidence interval; NE: not estimated; RBS: red breast syndrome.

**Table 4:** Patient baseline demographics and concurrent treatments.

	Reverse (-)	Reverse (+)	*p* value
No. of patients	14	9	
Age (yr)			0.336
Mean ± SD	45.88 ± 7.48	49.33 ± 8.11	
Range	36-62	32-59	
BMI (kg/m^2^)			0.557
Mean ± SD	22.2 ± 3.2	21.58 ± 3.11	
Range	15.88-26.56	16.08-28.37	
Smoking (*n*, %)	0	0	
Obesity (*n*, %)	0	0	
Radiotherapy (*n*, %)	3 (21.43%)	0	0.253
Preoperative	0	0	
Postoperative	3 (21.43%)	0	0.235
Chemotherapy (*n*, %)	6 (42.86%)	1 (11.11%)	0.176
Preoperative	2 (14.29%)	0	0.502
Postoperative	5 (35.71%)	1 (11.11%)	0.34
Pre+post	1 (7.14%)	0	>0.999

ADM: acellular dermal matrix; SD: standard deviation; BMI: body mass index. Obesity was defined as BMI > 25 kg/m^2^.

**Table 5:** Surgery-related variables.

	Reverse (-)	Reverse (+)	*p* value
No. of breasts	14	9	
Implant size (cc)			0.282
Mean ± SD	254.642 ± 83.526	216.667 ± 61.644	
Range	125-400	130-300	
Mastectomy weight (g)			0.361
Mean ± SD	283.429 ± 149.942	225.667 ± 81.974	
Range	55-547	90-342	
ADM size (m^2^)			0.625
Mean ± SD	121.143 ± 20.698	117.333 ± 41.569	
Range	80-160	64-192	
Jackson-Pratt drain (mL)			>0.999
Mean ± SD	580.243 ± 342.052	524.667 ± 99.919	
Range	126-1588.4	336-637	
Time to drain removal (day)			0.734
Mean ± SD	15.214 ± 3.051	15.556 ± 3.712	
Range	12-22	11-20	
Operation site (*n*, %)			0.68
Left	8 (57.14%)	4 (44.44%)	
Right	6 (42.86%)	5 (55.56%)	
Mastectomy type (*n*, %)			>0.999
NSM	7 (50.0%)	5 (55.56%)	
SSM	7 (50.0%)	4 (44.44%)	
ALND (*n*, %)	3 (21.43%)	2 (22.22%)	>0.999
Insertion plane (*n*, %)			0.343
Prepectoral	12 (85.71%)	6 (66.67%)	
Subpectoral	2 (14.29%)	3 (33.33%)	
Implant texture (*n*, %)			0.232
Smooth	13 (92.86%)	8 (88.89%)	
Microtexture	1 (7.14%)	1 (11.11%)	
Macrotexture	0	0	

SD: standard deviation; ADM: acellular dermal matrix; ALND: axillary lymph node dissection; SSM: skin sparing mastectomy; NSM: nipple areolar skin sparing mastectomy.

**Table 6:** Postoperative complications.

	Reverse (-)	Reverse (+)	OR^∗^	95% CI	*p* value
Total complication (*n*, %)	1 (7.14%)	2 (22.22%)	0.269	0.021-3.519	0.538
Minor^∗^	1	2			
Major^∗^	0	0			
Capsular contracture	0	0	NE		
Rippling	0	2 (22.22%)	NE		
Nipple sloughing	0	0	NE		
Rotation	0	0	NE		
Animation	0	0	NE		
RBS	1 (7.14%)	0	NE		
Mastectomy flap necrosis	0	0	NE		
Seroma	0	0	NE		
Hematoma	0	0	NE		

^∗^Major: the complications which needed secondary surgical procedures. ^∗^Minor: the complications which did not need secondary surgical procedures. ADM: acellular dermal matrix; OR: odds ratio; CI: confidence interval; NE: not estimated; RBS: red breast syndrome.

## Data Availability

The data used to support the findings of this study are restricted by the Asan Medical Center Institutional Review Board in order to protect patient privacy. Data are available from HH Han, tripleh1952@gmail.com, for researchers who meet the criteria for access to confidential data.
